# Safety and efficacy of convalescent plasma for severe COVID-19: a randomized, single blinded, parallel, controlled clinical study

**DOI:** 10.1186/s12879-022-07560-7

**Published:** 2022-06-27

**Authors:** Manuel Rojas, Yhojan Rodríguez, Juan Carlos Hernández, Juan C. Díaz-Coronado, José Alejandro Daza Vergara, Verónica Posada Vélez, Jessica Porras Mancilla, Iván Araujo, Jairo Torres Yepes, Oscar Briceño Ricaurte, Juan Mauricio Pardo-Oviedo, Diana M. Monsalve, Yeny Acosta-Ampudia, Carolina Ramírez-Santana, Paula Gaviria García, Lina Acevedo Landinez, Luisa Duarte Correales, Jeser Santiago Grass, Cristian Ricaurte Pérez, Gustavo Salguero López, Nataly Mateus, Laura Mancera, Ronald Rengifo Devia, Juan Esteban Orjuela, Christian R. Parra-Moreno, Andrés Alfonso Buitrago, Inés Elvira Ordoñez, Claudia Fabra Osorio, Nathalia Ballesteros, Luz H. Patiño, Sergio Castañeda, Marina Muñoz, Juan David Ramírez, Paul Bastard, Adrian Gervais, Lucy Bizien, Jean-Laurent Casanova, Bernardo Camacho, Juan Esteban Gallo, Oscar Gómez, Adriana Rojas-Villarraga, Carlos E. Pérez, Rubén Manrique, Rubén D. Mantilla, Juan-Manuel Anaya

**Affiliations:** 1 School of Medicine and Health Sciences, Doctoral Program in Biological and Biomedical Sciences, Universidad del Rosario, Bogota, Colombia; 2 Division of Rheumatology, Allergy and Clinical Immunology, University of California, Davis, Davis, CA USA; 3 Center for Autoimmune Diseases Research (CREA), School of Medicine and Health Sciences, Universidad del Rosario, Bogota, Colombia; 4 Clínica del Occidente, Bogota, Colombia; 5 Internal Medicine Department, Universidad CES, Medellin, Colombia; 6 Hospital Universitario Mayor –Méderi, Universidad del Rosario, Bogota, Colombia; 7 Clinica CES, Medellin, Colombia; 8 Instituto Distrital de Ciencia Biotecnología E Investigación en Salud, IDCBIS, Bogota, Colombia; 9 Centro de Investigaciones en Microbiología Y Biotecnología-UR (CIMBIUR), Facultad de Ciencias Naturales, Universidad del Rosario, Bogotá, Colombia; 10 Molecular Microbiology Laboratory, Department of Pathology, Molecular and Cell-Based Medicine, Icahn School of Medicine at Mount Sinai, New York, NY 10029 USA; 11 St Giles Laboratory of Human Genetics of Infectious Diseases, Rockefeller Branch, The Rockefeller University, New York, NY USA; 12 Laboratory of Human Genetics of Infectious Diseases, Necker Branch, INSERM, Necker Hospital for Sick Children, Paris, France; 13 University of Paris, Imagine Institute, Paris, France; 14 Department of Pediatrics, Necker Hospital for Sick Children, Paris, France; 15 Laboratory of Human Genetics of Infectious Diseases, Necker Branch, INSERM U1163, Necker Hospital for Sick Children, Paris, France; 16 University of Paris, Imagine Institute, Paris, France; 17 Department of Pediatrics, Necker Hospital for Sick Children, Paris, France; 18 Howard Hughes Medical Institute, Paris, France; 19 Genoma CES, Universidad CES, Medellin, Colombia; 20 Fundación Universitaria de Ciencias de la Salud (FUCS), Bogota, Colombia; 21 Infectious Diseases, Clínica de Marly, 110231 Bogotá, Colombia; 22 Epidemiology and Biostatistics Research Group, Universidad CES, Medellin, Colombia; 23 Present Address: LifeFactors, Rionegro, Colombia

**Keywords:** Clinical trial, SARS-CoV-2, COVID-19, Convalescent plasma

## Abstract

**Background:**

Convalescent plasma (CP) has been widely used to treat COVID-19 and is under study. However, the variability in the current clinical trials has averted its wide use in the current pandemic. We aimed to evaluate the safety and efficacy of CP in severe coronavirus disease 2019 (COVID-19) in the early stages of the disease.

**Methods:**

A randomized controlled clinical study was conducted on 101 patients admitted to the hospital with confirmed severe COVID-19. Most participants had less than 14 days from symptoms onset and less than seven days from hospitalization. Fifty patients were assigned to receive CP plus standard therapy (ST), and 51 were assigned to receive ST alone. Participants in the CP arm received two doses of 250 mL each, transfused 24 h apart. All transfused plasma was obtained from "super donors" that fulfilled the following criteria: titers of anti-SARS-CoV-2 S1 IgG ≥ 1:3200 and IgA ≥ 1:800 antibodies. The effect of transfused anti-IFN antibodies and the SARS-CoV-2 variants at the entry of the study on the overall CP efficacy was evaluated. The primary outcomes were the reduction in viral load and the increase in IgG and IgA antibodies at 28 days of follow-up. The per-protocol analysis included 91 patients.

**Results:**

An early but transient increase in IgG anti-S1-SARS-CoV-2 antibody levels at day 4 post-transfusion was observed (Estimated difference [ED], − 1.36; 95% CI, − 2.33 to − 0.39; P = 0.04). However, CP was not associated with viral load reduction in any of the points evaluated. Analysis of secondary outcomes revealed that those patients in the CP arm disclosed a shorter time to discharge (ED adjusted for mortality, 3.1 days; 95% CI, 0.20 to 5.94; P = 0.0361) or a reduction of 2 points on the WHO scale when compared with the ST group (HR adjusted for mortality, 1.6; 95% CI, 1.03 to 2.5; P = 0.0376). There were no benefits from CP on the rates of intensive care unit admission (HR, 0.82; 95% CI, 0.35 to 1.9; P = 0.6399), mechanical ventilation (HR, 0.66; 95% CI, 0.25 to 1.7; P = 0.4039), or mortality (HR, 3.2; 95% CI, 0.64 to 16; P = 0.1584). Anti-IFN antibodies and SARS-CoV-2 variants did not influence these results.

**Conclusion:**

CP was not associated with viral load reduction, despite the early increase in IgG anti-SARS-CoV-2 antibodies. However, CP is safe and could be a therapeutic option to reduce the hospital length of stay.

*Trial registration* NCT04332835

**Supplementary Information:**

The online version contains supplementary material available at 10.1186/s12879-022-07560-7.

## Introduction

The current pandemic has challenged health systems given the uncontrolled spread and high mortality of critically ill patients with coronavirus disease 2019 (COVID-19). Convalescent plasma (CP) emerged as a potential treatment for COVID-19 at the beginning of the pandemic [1]. This passive immunization strategy has been used to prevent and manage infectious diseases since the early twentieth century. This strategy has been previously implemented to treat several viral infections such as Spanish influenza, parvovirus B19, H1N1, Ebola, and other coronaviruses [1].

Some studies, including ours, showed that CP modulates the inflammatory response during acute COVID-19 [2–4]. The CP decreased activated and effector T cells and the IL-6/IFN-γ and IL-6/IL-10 ratios while increasing memory immune cells [2]. This was further confirmed by an additional study in which modulation of IP-10 and IL-6 was associated with improving hypoxia after CP administration [4]. Most clinical studies conducted during the pandemic confirmed that CP was implicated in reducing inflammatory markers, which could be associated with better clinical outcomes [5].

Despite the current evidence on the likely beneficial effects of CP for the treatment of COVID-19 via immunomodulation, a meta-analysis of randomized controlled trials (RCTs) showed that CP was not associated with a reduction in mortality [6]. However, current evidence must be taken with caution. Most published studies exhibited high methodological variability in selection criteria for donors and recipients, dosage, neutralizing antibodies (NAbs) concentration, disease severity, and outcomes, disclosing a high risk of bias [7]. On the other hand, a substantial negative correlation between CP use and mortality per admission in the United States offered population-level evidence that CP decreases mortality in COVID-19 and that the drop in utilization may have led to additional fatalities [8].

In this line, the variability between real-world scenarios and controlled clinical trials suggests that uncovered factors could influence the estimated efficacy of CP. Anti-IFN antibodies are implicated in mortality during acute COVID-19 [9]. However, whether passive transfusion of these autoantibodies by CP could harbor deleterious effects on recipients is unknown. In addition, as the pandemic evolved, several variants emerged with unpredictable repercussions on the estimated efficacy of CP in acute COVID-19.

Herein, we aimed to evaluate the safety and efficacy of CP in severe patients with COVID-19. The central hypothesis of this trial was that in patients with severe COVID-19, treatment with CP would be associated with a reduction of viral load and an increase in antibodies against severe acute respiratory syndrome coronavirus 2 (SARS-CoV-2) at 28 days. In addition, we explored the likely effect of transfused anti-IFN antibodies and the SARS-CoV-2 variants at the entry of the study on the overall CP efficacy.

## Methods

### Trial design

This study was a single-blinded, controlled, randomized, multicenter trial conducted at three clinical centers in Colombia: (1) Clínica de Occidente; (2) Clínica CES; and (3) Hospital de Méderi. The study recruitment was from August 8th, 2020, to November 13th, 2020. The follow-up was completed on December 11th, 2020. Eligible participants were randomly assigned in a 1:1 ratio to receive either CP and standard therapy (ST) or ST alone (i.e., defined by institutional protocols). The institutional review board of the Universidad del Rosario approved the study design (Act. 421 CEI-UR). Written informed consent was obtained from all participants, and the trial was conducted following the principles stated in the Declaration of Helsinki and Good Clinical Practice guidelines. The protocol was registered under the NCT04332835 clinical trials number.

### Inclusion and exclusion criteria

Inclusion criteria comprised the following: (1) signed informed consent; (2) aged at least 18 years; (3) COVID-19 diagnosis based on reverse transcriptase-polymerase chain reaction (RT-PCR) testing; (4) hospitalized patients; (5) Sequential Organ Failure Assessment score (SOFA) < 6 [10]; (6) severe cases according to Pneumonia Diagnosis and Treatment Scheme for Novel Coronavirus Infection (Trial Version 7) [11]. Severe COVID-19 was defined as respiratory distress with the following criteria: ≥ 30 breaths/minute in a resting state, oxygen saturation of 90% or less on room air, or arterial partial pressure of oxygen (PaO_2_)/fraction of inspired oxygen (FiO_2_) of 300 or less.

Exclusion criteria comprised the following: (1) pregnancy or breastfeeding; (2) patients with prior allergic reactions to transfusions; (3) critically ill patients in an intensive care unit (ICU): patients with requirement of ICU defined by the clinician, requirement of vasopressors, or mechanical ventilation; (4) patients with surgical procedures in the last 30 days; (5) subjects with active treatment for cancer (i.e., radiotherapy or chemotherapy); (6) diagnosed HIV in subjects with viral failure (i.e., detectable viral load > 1000 copies/mL, two consecutive viral load measurements within a 3-month interval, with medication adherence between measurements after at least six months of starting a new regimen of antiretrovirals); (7) subjects with a confirmed infection that explains clinical manifestations; (8) end-stage kidney disease (i.e., glomerular filtration rate < 15 ml/min/1.73 m^2^); (9) Child–Pugh C stage liver cirrhosis; (10) high cardiac output diseases; (11) autoimmune diseases or Immunoglobulin A nephropathy; and (12) subjects not willing to sign a written informed consent. The time frame was not considered an exclusion or inclusion criteria for the study. However, all the patients received transfusions within the first seven days of hospitalization.

### Randomization, masking and blinding

The study investigators screened potential study participants for eligibility prior to randomization. A maximum lapse of 24 h was allowed for the screening and consent process. A completely blocked randomized design to form the allocation list for the two comparison groups. A computer random number generator was used to build random permuted blocks with a block size of four and an equal allocation ratio. The randomization process was carried out by the designated blinded investigator, who did not acknowledge the treatment assignment. The study statistics team was also blinded to elaborate interim analysis and safety reports. Both participants and the clinical research team were unblinded to the treatment assignment.

### Procurement of convalescent plasma

The pre-donation process included the following steps/criteria: (1) signed informed consent; (2) aged between 18 and 65 years; (3) subjects with a laboratory-confirmed COVID-19 diagnosis by RT-PCR having been hospitalized but not at ICU, discharged, and recovered between 14 and 30 days before the pre-donation assessment; (4) two consecutive negative RT-PCR results from nasopharyngeal swabs within 48 h before donation; (5) women were only accepted if they did not have pregnancy history or a current suspicion of pregnancy; (6) negativity for HIV, hepatitis B and C virus, HTLV 1 and 2, syphilis, and *Trypanosoma cruzi* infection.

Furthermore, plasma values of IgG 1:3200 and IgA 1:800 for SARS-CoV-2 antibodies (by Enzyme-Linked ImmunoSorbent Assay—ELISA -, Euroimmun, Lübeck, Germany) were considered to be a "super donor" for plasmapheresis. As previously reported, these thresholds secured a minimal plaque reduction neutralization test (PRNT_50_) ≥ 1:256 [2]. Approximately 800 mL of plasma were collected from donors. Prior to freezing, pathogen inactivation with Riboflavin followed by UV light exposure was performed [12].

### Intervention

Data curators were unaware of the treatment assignments. Each transfusion dose of CP was 250 mL (i.e., patients received two doses for a total of 500 mL within 48 h of study inclusion). In most cases, the transfused CP ABO type was compatible with the recipient's ABO type. CP transfusion was administered at 3 ml/min with close monitoring for the first 30 min and regular monitoring over the following 6 h. ST consisted of symptomatic control and supportive care for COVID-19. This treatment was based upon recommendations from the Colombian Association of Infectology and institutional protocols, including management with antibiotics, corticosteroids, oxygen, or anticoagulants [13]. Both plasma recipients and ST groups received this treatment. None of the patients received experimental therapies, including tocilizumab, ivermectin, colchicine, antimalarials, or antivirals.

### Clinical outcomes

Primary outcomes were: (1) reduction of viral load and (2) increase in titers of IgG and IgA for SARS-CoV-2. Secondary outcomes were as follows: (1) proportion of patients with the requirement of ICU admission; (2) proportion of patients with the requirement of invasive mechanical ventilation; (3) length of hospital stay; (4) adverse events; (5) improvement of 2 points in the World Health Organization (WHO) ordinal scale for clinical improvement [14]; and (6) proportion of mortality. Primary and secondary outcomes were evaluated on days 4, 7, 14, and 28 after the infusion of CP, except for total antibodies, which were measured on days 4 and 28.

### Laboratory evaluation

The viral load was measured using the Ampliphi ® RT > -qPCR SARS-CoV-2 Viral Load Kit (www.ampliphi.co). The Euroimmun anti-SARS-CoV-2 ELISA (Euroimmun, Luebeck, Germany) was used for serological detection of human IgG and IgA antibodies against the SARS-CoV-2 S1 structural protein, in accordance with the manufacturer's instructions. The ratio interpretation was < 0.8 = negative, ≥ 0.8 to < 1.1 = borderline, ≥ 1.1 = positive [15–17]. Antibody titration was performed using serial dilutions of serum samples of 1:100 on days 0 and 1:200 on days 4 and 28. Anti-IFN antibodies were evaluated as previously described in all super donors and 97 out of 101 randomized patients [9].

### Whole genomes sequencing and analyses

Whole-genome sequencing of SARS-CoV-2 was performed using Oxford Nanopore's MinION platform, and the MinKNOW application (v1.5.5) according to the established protocol (https://artic.network/ncov-2019). The bioinformatics analysis was performed following the algorithm for MinION sequences described in the ARTIC bioinformatics pipeline (https://artic.network/ncov-2019/ncov2019-bioinformatics-sop.html). Once the assemblies were obtained, typing was performed for those genomes with at least 60% coverage and maximum 40% Ns based on the Pangolin COVID-19 Lineage Assigner (Phylogenetic Assignment of Named Global Outbreak LINeages) [18].

Comparative genomics analyses were developed to evaluate the phylogenomic relationships for the 43 genomes obtained in this study in the context of 2,170 genomes from a representative selection of all SARS-CoV-2 lineages obtained from NextClade tool v 1.5.4 (https://clades.nextstrain.org/). For this, the total dataset with 2,213 genomes was aligned and used to build a maximum likelihood (ML) in NextClade following the pipeline of analysis previously published [19].

A mutational analysis was performed over the 43 SARS-CoV-2 whole genome sequences obtained in this study using the NextClade tool. The presence and frequency of mutations were assessed for all the genomes using as reference the genome from Wuhan, China (hCoV-19/Wuhan/Hu-1/2019, GenBank accession number: NC_045512.2). Furthermore, the nucleotide variation among samples with different collection day after the treatment (standard or plasma) was compared, for the following five patients: (1) RC022, with three samples (collection day 0, 4 and 14 after a standard treatment); a second patient; (2) RC054, with two samples (collection day 0 and 4 after the plasma treatment); (3) RC058 with two samples (collection day 0 and 7 after a standard treatment); (4) RC076, with two samples (collection day 0 and 14 after a standard treatment); and (5) RC083 with two samples (collection day 0 and 7 after a standard treatment).

### Data collection and clinical follow-up

Demographics, comorbid conditions, and concomitant medications were recorded at enrollment. Castor EDC was used for data collection (***https://www.castoredc.com/). Patients were clinically followed for 28 days after enrollment. Clinical and paraclinical parameters were obtained using a standardized form. The former comprised all the variables that were included in the global COVID-19 clinical platform from the WHO [20]. The biological baseline (day 0) included viral load, blood gases, a laboratory surrogate of a possible thrombotic process (D-Dimer), hematological, inflammatory, hepatic, and renal parameters. These measurements were repeated on days 4, 7, 14, and 28. In the case of an earlier hospital discharge, the in-hospital follow-up was scheduled on days 4, 7, 14, and 28 to look for clinical outcomes and adverse events until day 28, in compliance with current healthcare protection policies. Adverse events were registered and reported on an ongoing basis.

### Sample size and statistical analysis

We included 92 patients, 46 in each group. The sample size calculation for the trial was performed using the STATA 16.0 software, in the binary outcomes sub-module and comparison of the results with the chi-square test, under a unilateral hypothesis test, using the following assumptions: α error: 0.05, Power (1-β): 0.90, minimum expected difference: 0.30, the proportion of patients with an unfavorable result in the experimental group: 0.35, the proportion of patients with an unfavorable outcome in the control group: 0.65, Ratio 1:1 between the experimental group and the control group. We considered a maximum expected proportion of losses: 0.25.

In the univariate analysis, categorical variables were analyzed using frequencies, and continuous quantitative variables were expressed as the mean and standard deviation (SD) or the median and interquartile range (IQR) according to the observed distribution. The Fisher exact tests or Mann–Whitney U-test were used based on the results.

Viral load was analyzed after log_10_ transformation; all other parameters were analyzed without additional data transformation. Due to the longitudinal nature of the study and in order to identify clinically and statistically essential characteristics, generalized linear mixed models (GLMMs) were used, as recommended [21–28]. Delta change of log_10_ viral load and the ratio of antibodies were analyzed with a linear mixed model for repeated measures, based on a covariance structure of variance components. To quantify effect sizes, their parameters were estimated using the Minimum Quadratic Unbiased Variance Estimation method.

$$g\left( {\mu_{ij} } \right) = \beta_{0} + \beta_{1} Group_{i} + \beta_{2} Day_{ij} + \beta_{3} Group_{i} *Day_{ij} + b_{i}$$

where $g({\mu }_{ij})$ is the link function, $i=\mathrm{1,2},\cdots ,n$ and $j=\mathrm{1,2},\cdots ,{n}_{i}$. Also, *b*_*i*_ is a random intercept which is helpful to model deviations from the mean model. For delta change of viral load and SARS-CoV-2 IgG and IgA ratios, post hoc comparison of means was based on both adjusted Bonferroni p-values and Fisher's protected least significant differences procedure using t statistics based on Satterwhaite's approximation. The same model was used for the probability of viral load negativization but based on the logistic link function.

A Cox proportional hazard regression model to evaluate time to death, time to ICU admission, time to MV, and time to clinical improvement to estimate the Hazard Ratios (HR) was used. The estimation of HR for clinical improvement was adjusted for mortality. All assumptions for these models were met. In addition, we implemented the Kaplan Meier method to estimate the cumulative incidence as a function of time. We conducted a linear model adjusted for mortality for days of hospitalization, and a post-hoc comparison between groups was estimated. All association measures were presented with 95% confidence intervals (95% CI). Subgroup analyses were not conducted. All analyses were performed with R version 4.0.1.

## Results

### Patients

**Fig. 1:**
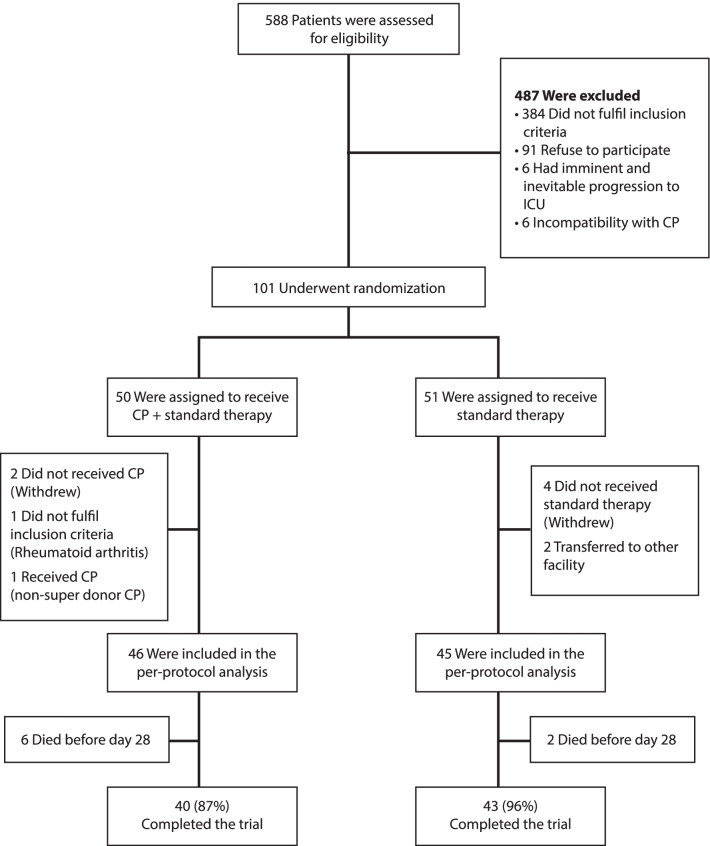
Enrollment and Randomization

Overall, 101 patients underwent randomization. Fifty patients were assigned to receive CP plus ST, and 51 were assigned to receive ST alone. We included all randomized patients in the analysis according to the randomization arm, except those who withdrew after randomization, were transferred to other facilities, a patient transfused with non-competent CP, and a patient with rheumatoid arthritis. The per-protocol analysis included a total of 91 patients (Fig. 1).

The general characteristics of patients are shown in Table 1. The median age of the patients and coexisting conditions at entry into the trial were similar between groups. Most of the patients included were men, and the therapeutics implemented on admission were comparable. The median time to inclusion from symptom onset was ten days, and the median time from hospital admission to plasma transfusion was 2.5 days. Oxygen saturation below 90% and a PaO_2_/FiO_2_ of 300 or less were the most common severity criteria for enrollment. All patients were receiving oxygen at the time of entry into the trial and presented a WHO scale score between 4 and 5, and the 4C Mortality and CHOSEN scores did not differ between groups. Levels of biomarkers for inflammation and organic dysfunction were similar.

**Table 1:** General Characteristics of Patients with COVID-19

Variable	Standard therapy (n = 45)	Plasma recipients (n = 46)	P value^a^
*Demographics*			
Sex (%)			0.8210
Female	14 (31.1)	13 (28.3)	
Male	31 (68.9)	33 (71.7)	
Age (Median—IQR)	54 (48—59)	55.5 (38—62.8)	0.8333
BMI (Median—IQR)	28.2 (26.1—30.5)	30.9 (27.8—35.2)	0.0011
*Therapy (%)*			
Corticosteroids	43 (95.6%)	45 (97.8%)	0.6166
Antibiotics	22 (48.9%)	22 (47.8%)	1.0000
NSAIDs	0 (0.0%)	1 (2.2%)	1.0000
Heparin	42 (93.3%)	45 (97.8%)	1.0000
*Clinical characteristics*			
WHO scale (%)			0.8202
4 points	30/44 (68.2)	32/45 (71.1)	
5 points	14/44 (31.8)	13/45 (28.9)	
Log viral load (Estimated mean—SE)	6.4 (0.57)	5.63 (0.58)	1.0000
4C Mortality score (Median—IQR)	6 (5 – 9)	7 (5.3 – 9)	0.4850
CHOSEN score (Median—IQR)	17 (16 – 31)	16.5 (10 – 23.8)	0.0965
SOFA on inclusion (Median—IQR)	2 (2—2)	2 (2—2)	0.3728
PaO_2_-FiO_2_ on inclusion (Median—IQR)	182.3 (123—233)	178 (111.6—249.7)	0.8728
Time from symptoms onset to inclusion (Days—Median—IQR)	10 (7 – 11)	10 (8 – 11)	0.2880
Time from symptoms onset to plasma transfusion (Days—Median—IQR)	-	11 (9 – 12)	-
Time from hospital admission to plasma transfusion (Days—Median—IQR)	-	2.5 (2 – 3)	-
*Comorbidities (%)*			
Hypertension	10 (22.2)	13 (28.3)	0.6308
Dyslipidemia	12 (26.7)	20 (43.5)	0.1248
Asthma	3 (6.7)	1 (2.2)	0.3610
CKD	3 (6.7)	2 (4.3)	0.6768
Acid-peptic disease	13 (28.9)	9 (19.6)	0.3357
Diabetes	6 (13.3)	9 (19.6)	0.5737
Current smoker	1 (2.2)	2 (4.3)	1.0000
Former smoker	19 (42.2)	20 (43.5)	1.0000
*Admission laboratories (Median—IQR)*			
Platelets × 10^3^ per cubic millimeter	258 (211—327)	282 (223—348)	0.5596
Leucocytes	10,230 (7800—13,960)	9235 (6922.5—11,540)	0.0747
Lymphocytes	750 (590—1050)	985 (752.5—1355)	0.0080
Neutrophils	9150 (6600—12,500)	7865 (5667.5—9837)	0.0354
C reactive protein (mg/L)	78.21 (21.6—121.8)	49.30 (14.21—111.82)	0.3718
Erythrocyte sedimentation rate (mm/hr)	30 (13—44)	25 (15—45)	0.6984
Albumin (g/dL)	3.5 (3.27—3.74)	3.40 (3.21—3.68)	0.2802
Total bilirubin (µmol/L)	0.49 (0.41—0.67)	0.46 (0.30—0.66)	0.1387
Urea (mg/dL)	18.9 (14.7 – 22.8)	15.75 (14.13—23.11)	0.4436
Creatinine (mg/dL)	0.81 (0.66—0.92)	0.82 (0.7—0.95)	0.4506
Creatine kinase (U/L)	80 (47—185)	93 (50—173.3)	0.5516
D-Dimer (mg/L)	0.65 (0.33—1.06)	0.64 (0.45—1.07)	0.6031
Ferritin (ng/mL)	1199 (727.9—1921)	1399 (858—2161.5)	0.6856
Lactate dehydrogenase (U/L)	356 (294—446)	373.5 (299—441.5)	0.7002
Procalcitonin (ng/mL)	0.17 (0.11—0.37)	0.2 (0.10—0.42)	0.9968
Troponin T (ng/mL)	0.007 (0.003—0.37)	0.009 (0.005—0.34)	0.7142

^a^ p values for categorical variables obtained by Fisher's exact test. Quantitative variables were analyzed by Mann–Whitney U-test. Differences of viral load at baseline were obtained from the post hoc analysis from the generalized linear mixed model. Abbreviations: BMI: Body mass index; NSAIDs: Non-steroidal anti-inflammatory drugs; SE: Standard error; SOFA: Sequential organ failure assessment; WHO: World health organization; IQR: Interquartile range; CKD: Chronic kidney disease

### Primary outcomes

**Fig. 2:**
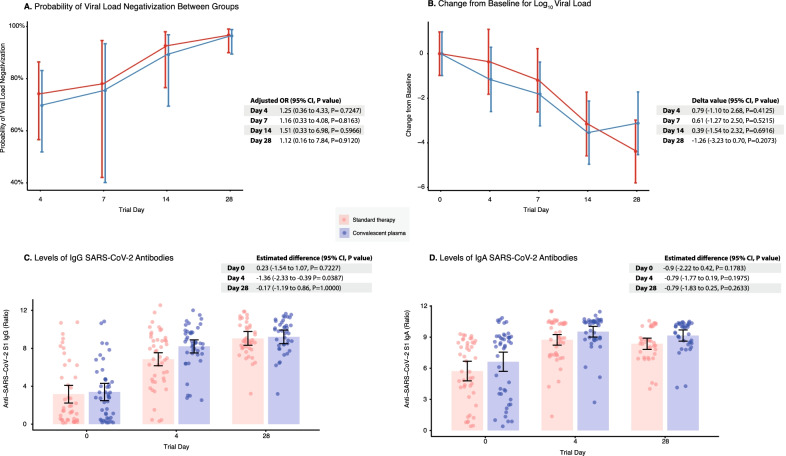
Primary outcomes. **A** Probability for Viral Load Negativization. **B** Change in Log10 Viral Load. **C** Change in anti-SARS-CoV-2 S1 IgG antibodies. **D** Change in anti-SARS-CoV-2 S1 IgA antibodies. OR: Odds ratio; CI: Confidence interval

Analysis of primary outcomes revealed that administration of CP was not associated with an increased probability of negativization or reduction of viral load during the 28-day follow-up (Fig. 2A, B). At baseline, IgG (Estimated difference [ED], − 0.23; 95% CI, − 1.54 to 1.07; P = 0.7227) and IgA (ED, − 0.9; 95% CI, − 2.22 to 0.42; P = 0.1783) anti-S1-SARS-CoV-2 antibodies were similar between groups. Then, CP administration was associated with an early but transient increase in IgG antibodies levels at day 4 post-transfusion (ED, − 1.36; 95% CI, − 2.33 to − 0.39; P = 0.0387), but not in IgA antibodies (ED, − 0.79; 95% CI, − 1.77 to 0.19; P = 0.1975) (Fig. 2C). After 28 days of follow-up, IgG and IgA antibodies levels were similar between the two arms of treatment (Fig. 2D).

### Secondary outcomes

**Fig. 3:**
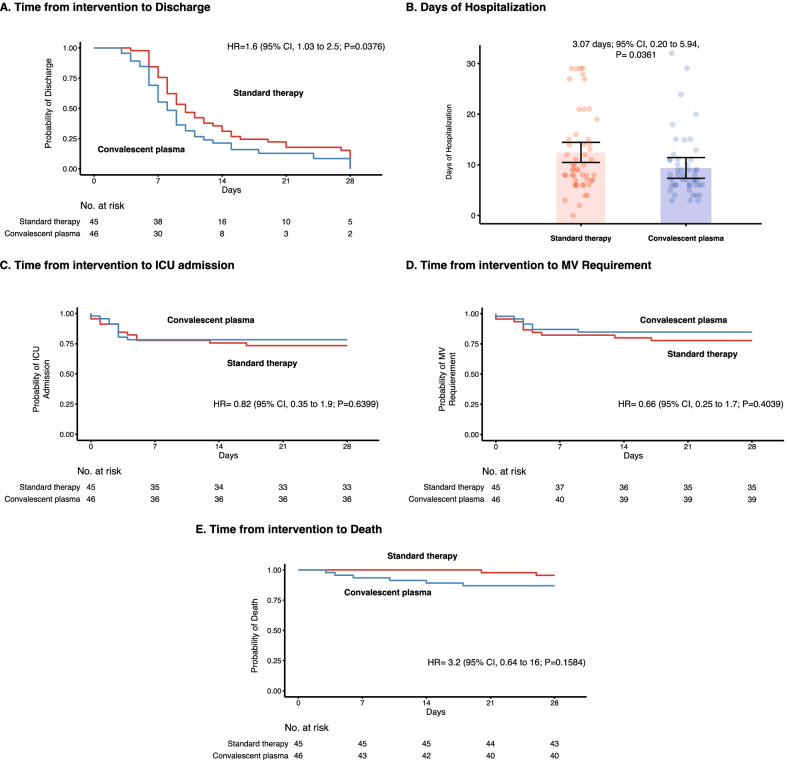
Secondary outcomes. Kaplan–Meier Estimates for **A** Time to ICU Admission. **B** MV Requirement. **C** Death. **D** Hospital Discharge. **E** Estimated Differences in Days of Hospitalization Adjusted for Mortality. MV: Mechanical ventilation; ICU: Intensive care unit; CI: Confidence interval

Analysis of secondary outcomes revealed that the CP arm discloses a shorter time to discharge or reduction in 2 points in the WHO ordinal scale compared with the ST arm (HR adjusted for mortality, 1.6; 95% CI, 1.03 to 2.5; P = 0.0376) (Fig. 3A). Mean hospitalization days in the CP arm was 11.1 (95% CI, 8.3 to 14.0), while in the ST arm, it was 14.2 days (95% CI, 11.1 to 17.3) (ED adjusted for mortality, 3.1 days; 95% CI, 0.20 to 5.94; P = 0.0361) (Fig. 3B). ICU admission was observed in 10 patients (22%) who received CP, and in 12 patients who received ST (27%) (HR, 0.82; 95% CI, 0.35 to 1.9; P = 0.6399). Mechanical ventilation was registered in 7 (15%) patients who received CP, and in 10 (22%) who received ST (HR, 0.66; 95% CI, 0.25 to 1.7; P = 0.4039). Death was observed in 6 and 2 patients who received CP (13%) and ST (4%), respectively (HR, 3.2; 95% CI, 0.64 to 16; P = 0.1584) (Fig. 3C–E).

### Safety

Only 1 (2.17%) infusion-related adverse event was observed in the CP group (mild rash with pruritus and palpebral edema). There were no differences in the overall incidence of adverse events (odds ratio [OR], 0.87; 95% CI, 0.32 to 2.36) or serious adverse events (OR, 0.78; 95% CI, 0.27 to 2.18) (Table 2).

**Table 2:** Safety of convalescent plasma in the per-protocol analysis

Adverse events	Standard therapy (n = 45)	Plasma recipients (n = 46)	OR (95% CI)
Overall	14 (31.1%)	13 (28.3%)	0.87 (0.32 to 2.36)
*Serious*	13 (28.9%)	11 (23.9%)	0.78 (0.27 to 2.18)
MV	10 (22.2%)	7 (15.2%)	–
ICU admission	0 (0.0%)	2 (4.4%)	–
Pulmonary embolism	1 (2.2%)	1 (2.2%)	–
DVT	0 (0.0%)	1 (2.2%)	–
Pneumatocele	1 (2.2%)	0 (0.0%)	–
Readmission	1 (2.2%)	0 (0.0%)	–
*Non-serious*^a^	1 (2.2%)	2 (4.4%)	2.0 (0.10 to 120.54)
Rash with pruritus	1 (2.2%)	1 (2.2%)	–
Palpebral edema	0 (0.0%)	1 (2.2%)	–

^a^ A patient presented rash with pruritus and palpebral edema after administration of CP (related adverse event). CP: Convalescent plasma; DVT: Deep venous thrombosis; ICU: Intensive care unit; MV: Mechanical ventilation; OR: Odds ratio; CI: Confidence interval

### IFN autoantibodies

From 21 super donors recruited for the study, one (4.8%) presented neutralization activity for anti-IFNα and two (9.5%) for anti-IFNω. Two patients who received plasma with these autoantibodies survived and did not present adverse outcomes during the follow-up. In addition, neutralizing activity at the entry of the clinical trial for anti-IFNα and anti-IFNω was found in 5/97 (5.2%) and 3/97 (3.1%) patients, respectively. None of the patients with these autoantibodies were admitted to ICU, required mechanical ventilation, or died.

### SARS-CoV-2 variants

A total of 43 SARS-CoV-2 genomes from 32 patients (12 and 20 patients from CP and ST groups, respectively) were sequenced, assembled, and classified through the lineage assigner Pangolin. From the 43 sequences, 41.9% was classified as B.1, followed by the lineages B.1.420, B.1.111, B.1.177.73, and B.1.465, with a proportion of 25.6%, 23.3%, 2.3%, and 2.3%, respectively. Additionally, the lineage P.1, also known as the Variant of Concern (VOC) Gamma, was found in the analysis performed with a proportion of 4.7% (Additional file 1: Table S1A).

**Fig. 4:**
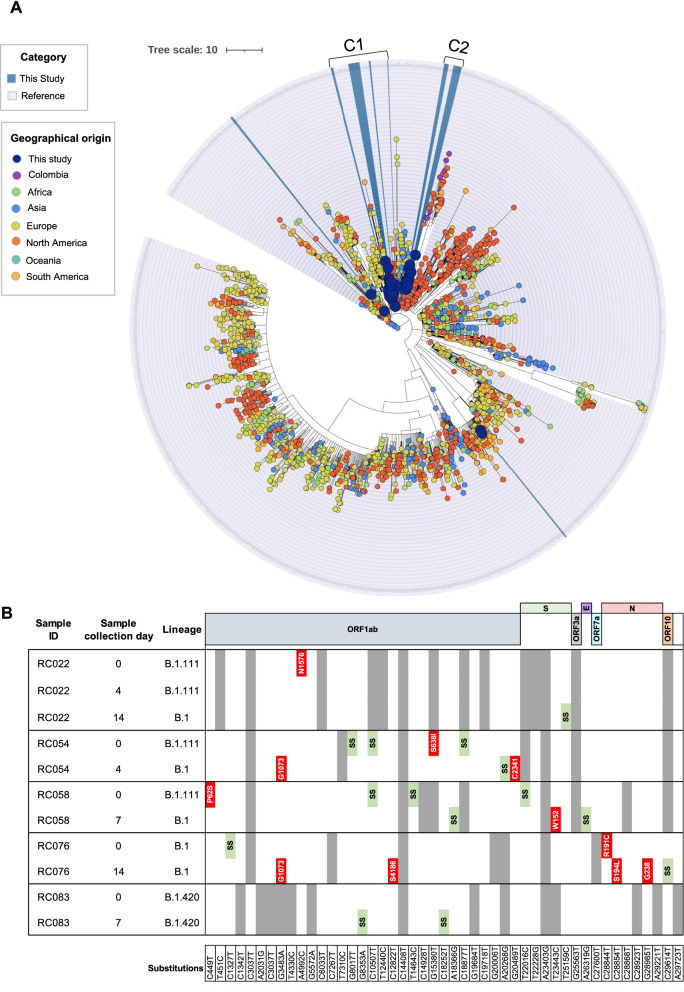
Comparative genomics and nucleotide diversity analyses among SARS-CoV-2 genomes obtained in this study. **A** Phylogenomic relationships using a maximum likelihood phylogeny obtained in Nextclade. **B** Whole-genome substitutions profiles for the eleven genomes from five patients with more than one sample. Mismatches were identified by comparison with the Wuhan reference sequence (NC_045512.2). The substitutions shared among each group of samples are represented in grey and the substitutions found on any of the sample's days are represented in Red (non-synonymous substitutions) or light green (SS: synonymous substitutions). Inside of each green box is described the type of substitutions

Most of the genomes analyzed in this study (n = 38/43) were grouped into two main monophyletic clusters that were labeled C1 and C2 (Fig. 4A). C1 included 21 genomes that were mainly classified as B.1.420 lineage and were collected from Bogota. This cluster was closed to reference genomes from Colombia and South America. Otherwise, C2 included 17 genomes belonging mostly to B.1.111 lineage with an equitable origin distribution between Bogota and Medellin (Fig. 4A).

Initially, we evaluated mutations of interest in the 43 samples analyzed. The results showed mutations mainly in the region encoding the spike protein. The 100% of sequences presented the mutation D614G, followed by the E484K, N501Y, and Q677H mutations in a proportion of 2.3%. Finally, the deletion of four nucleotides (del: 26,158:4) was identified, also in the spike protein (Additional file 1: Table S1B).

We identified substitutions in the five patients in whom samples were obtained for genotyping before and after treatment (Fig. 4B). For patient 1 (RC022, ST group), we identified sixteen substitutions; fourteen were shared among all the days sampled, and two were found on only one of the days. On day zero, one substitution was identified, located in the ORF1ab (A4992C: N1576T), and on day 14, an additional substitution in the Spike (T25159C). Fourteen substitutions were identified in patient 2 (RC054, CP group). Seven were found in only one day sampled; four on day zero (ORF1ab: G8017T; C10507T; G15380T: S638I and C18877T) and three on day 4 (ORF1ab: G3483A: G1073E, A20268G, and G20489T: C2341F).

Regarding patient 3 (RC058, ST group), sixteen substitutions were identified. Nine were shared among all the days sampled, and seven substitutions were found in only one of the days sampled but not on the others. On day zero, four substitutions were found (three on the ORF1ab (C449T: P62S; C10507T and T14643C) and one in the S (T22016C)) and three substitutions on day 7 (one on the ORF1ab: A18366G; one in the S: T23443C: W152R and one in the E: A26319G). For the case of patient 4 (RC076, ST group); seven substitutions were found in only one of the sampled days, two of them identified on day zero (ORF1ab: C1327T and N: C28844T: R191C) and five on day 14 (ORF1ab: G3483A: G1073E and C12822T: S4186F; N: C28854T: S194L and G28985T: G238C and ORF10: C29614T). Finally, patient 5 (RC083, ST group) revealed sixteen substitutions, two of them were found only on day 7: ORF1ab G8353A and C18252T (Fig. 4B).

There were no differences in the distribution of variants by treatment (Fisher's exact test, P = 0.8258). In addition, none of the variants were associated with ICU admission (Fisher's exact test, P = 1.0000), mechanical ventilation (Fisher's exact test, P = 0.8659), or death (Fisher's exact test, P = 0.0916).

## Discussion

In this study, conducted on severe COVID-19 patients, CP was associated with a shorter hospital stay and early clinical recovery. As expected, CP was associated with higher levels of IgG antibodies post-transfusion, but this phenomenon was not associated with viral load reduction. Mortality, the requirement of ICU, and mechanical ventilation were not modified by CP administration. None of these results seemed to be influenced by anti-IFN antibodies or the SARS-CoV-2 variants.

It has been suggested that administration of a high titer of anti-SARS-CoV-2 IgG antibodies could be associated with better clinical outcomes in COVID-19 [29]. However, none of the published RCTs have determined the minimum CP concentration of IgG or IgA antibodies to substantially increase these antibodies in the recipients. Simonovich et al. [30], showed that patients transfused with CP exhibited a slight increase in titers of antibodies at two days post-transfusion compared with placebo. However, this trend did not reach statistical significance. Both groups showed similar levels of anti-SARS-CoV-2 antibodies after 7 and 14 days of follow-up [30]. Patients from that study received a minimum of 1:800 IgG SARS-CoV-2 antibodies [30]. In our study, CP containing ≥ 1:3200 IgG antibodies significantly increased anti-SARS-CoV-2 post-transfusion but lasted less than 4 days.

Data from observational studies suggested that CP induced a reduction in viral load [31, 32]. The first RCT showed that patients had negativization of viral load in the first 72 h post-transfusion [33]. However, other studies showed no effect of CP on viral load kinetics [34, 35]. In ours, CP did not modify the probability of viral load negativization or viral load reduction in RNA copies per swab despite the high IgG and IgA antibodies transfused. This is of interest since the primary mechanism of action of CP in COVID-19 is thought to be related to SARS-CoV-2 neutralization. Thus, multidose regimens could be necessary to maintain antibody levels and achieve adequate viral clearance.

Concerning the lineages that affected patients, the Pangolin lineage classifier showed that the 43 genomes sequenced were clustered in six main lineages. The majority were classified as B.1 (Additional file 1: Table S1A), lineage primarily reported in Europe and South America and with an origin associated with a Northern Italian outbreak early in 2020 [36]. Additionally, we describe the diversity dynamics of the virus by identifying sub-lineages from the B.1 parental lineage, and the presence of a variant widely associated with the increased transmissibility, major viral load, immunological escape, and the capacity of reinfections, as VOC Gamma [37]. The phylogenomic analysis revealed the existence of clusters C1 and C2 that corresponded to the most abundant lineages B.1.420 and B.1.111, respectively (Fig. 4A). These findings were consistent with the lineage distribution profiles reported for Colombia in the analyzed timeline [19]. Interestingly, C2 (B.1.111) had a heterogeneous distribution in the regions of origin (Bogota and Medellin), which shows a loss of geographic delimitation of this lineage with a high global distribution. Finally, analyzing the substitutions in the 43 genomes sequenced, we found not only polymorphisms previously reported in the spike gene (i.e., E484K, N501Y, and Q677H) (Additional file 1: Table S1B), which could impact the transmission rate and the effectivity of CP, due to capacity to generate immunological escape [18], but also the presence of a mutational profile (Fig. 4B), in each of the lineages identified. These results are consistent with previous reports [38–40]. The appearance of new SARS-CoV-2 mutations in the same variants throughout the study reinforces the high viral mutation capacity observed in both groups of patients. Further and larger studies are required to evaluate the evolutionary forces associated with this phenomenon.

Most of the published studies exhibited high methodological variability in selection criteria for donors and recipients, dosage, NAbs titers, disease severity, and outcomes [7]. Thus, it is difficult to establish the real therapeutic effect of CP over outcomes such as mortality, mechanical ventilation, or ICU admission. None of the early RCTs published showed benefits in these outcomes in hospitalized [30, 33–35, 41–45], or ambulatory patients [46]. Similarly, the RECOVERY trial that was stopped early since high CP titers did not improve survival in hospitalized COVID-19 patients [47]. However, as the pandemic evolved, further RCTs showed that the administration of CP in the early stages of the disease and elderly patients was beneficial [48, 49]. Although we found a slight increase in mortality in patients who received CP, the likelihood of this outcome was not significant. A recent meta-analysis in which 16.477 patients were included indicated that CP is not associated with increasing or reducing mortality rates [6]. However, this evidence must be taken with caution since recent population-based data support its utilization in the early stages of the disease with favorable effects [8]. Further population-based studies may help confirm its real therapeutic effect and the role of CP from vaccinated donors.

In our study, patients treated with CP demonstrated faster clinical recovery and shorter hospital stay (i.e., 3.07 days less hospitalization). This is in line with the results from Ray et al. [34] that found a shorter hospital stay in patients treated with CP (i.e., 4 days). A recent systematic review on CP showed that about 20% of the studies had found a reduction in hospital length of stay [5]. All the data mentioned above suggest that despite the lack of influence on mortality, CP may help to reduce the burden of the disease on the health system. This deserves further cost-effective analysis to critically evaluate the introduction of CP as initial therapy in hospitalized patients with COVID-19.

The paradoxical effect of CP on early recovery without an effect on mortality suggests that personalized selection of recipients could improve its efficacy. Park et al. [50] showed that patients with preexisting conditions (diabetes, cardiovascular and pulmonary diseases), blood type A or AB, and at an early stage of COVID-19 (low baseline WHO scores) were expected to benefit the most. In contrast, those without preexisting conditions and at later stages of COVID-19 could potentially be harmed. This strategy may help increase the effectiveness of CP in real-life situations and allow new clinical trials to be designed.

New-onset autoantibodies have been found in acute COVID-19 [51], and latent polyautoimmunity seems to influence deleterious outcomes in hospitalized patients [52]. In addition, anti-IFN antibodies are implicated in mortality and correlate with age [9]. Since most of the collected plasma in our study was from patients with prior history of hospitalization, we evaluated the frequency and the effect of anti-IFN antibodies on clinical outcomes. We found similar frequencies of positivity to those reported in the literature (i.e., ~ 5%) [9, 53]. In addition, we found that transfused anti-IFN antibodies did not influence the clinical outcomes. Raadsen et al.[54] showed that anti-IFN-α2 antibodies in COVID-19 CP donors were not neutralizing, thus suggesting that despite the positivity of ELISA, these antibodies may have a low influence on outcomes once they are transfused. However, given the low frequency of these autoantibodies in this cohort, larger studies are needed to confirm that anti-IFN antibodies do not affect CP recipients with COVID-19.

Our study has several strengths. We included patients in the first 72 h of admission and in the first 14 days after symptoms onset, which allowed evaluating the effects of CP in the early stages of the disease. The timing of inclusion guaranteed comparability in clinical progression and disease staging. All included patients fulfilled the definition of severe COVID-19. In addition, transfused patients received high-titer CP that surpassed the IgG anti-SARS-CoV-2 antibodies used in prior RCTs. None of the included patients received experimental treatments that may have biased the estimations obtained in this study.

Limitations must be acknowledged. This was a single-blinded trial. Thus, it was susceptible to bias in outcome ascertainment by clinical doctors. In addition, the measurement of viral load may have been influenced by technical factors that did not allow an accurate measurement of this outcome. However, the analysis revealed that viral load decreased in both groups equally, suggesting that our results replicate the natural history of viral load in COVID-19. There were no unexpected SARS-CoV-2 variants in the analyzed timeline. Thus, results should be interpreted within this scenario. It is worth mentioning that studies on immunocompromised patients have shown clinical benefits [55, 56]. However, we excluded this group of patients.

## Conclusions

This study found that CP reduced hospital length of stay but not viral load, ICU requirement, mechanical ventilation, or mortality. Further cost-effective analyses are required to evaluate the inclusion of CP as additional therapy to the conventional one.

## Supplementary Information

**Additional file 1.** Lineages and mutations of interest in the studied population.

## Data Availability

Sequences and metadata of SARS-CoV-2 genomes generated during the current study for genomic analyses are available in the Github repository https://github.com/gimur/CREA_Paper. We thank the contributions of both the submitting and the originating laboratories who participated in the Global Initiative for Sharing All Influenza Data (GISAID) database as contributors to the public sequences used for comparative genomics analyses.

## Abbreviations

COVID-19: Coronavirus disease 2019
CP: Convalescent plasma
ED: Estimated difference
FiO_2_: Fraction of inspired oxygen
GLMMs: Generalized linear mixed models
HR: Hazard Ratios
ICU: Intensive care unit
IQR: Interquartile range
MV: Mechanical ventilation
Nabs: Neutralizing antibodies
OR: Odds ratio
PaO_2_: Partial pressure of oxygen
PRNT_50_: Plaque reduction neutralization test
RCTs: Randomized controlled trials
RT-PCR: Reverse transcriptase-polymerase chain reaction
SARS-CoV-2: Severe acute respiratory syndrome coronavirus 2
SD: Standard deviation
SOFA: Sequential Organ Failure Assessment score
ST: Standard therapy
VOC: Variant of Concern
WHO: World Health Organization

## References

[1] Rojas M, Rodríguez Y, Monsalve DM, Acosta-Ampudia Y, Camacho B, Gallo JE (2020). Convalescent plasma in Covid-19: Possible mechanisms of action. Autoimmun Rev.

[2] Acosta-Ampudia Y, Monsalve DM, Rojas M, Rodríguez Y, Gallo JE, Salazar-Uribe JC (2021). COVID-19 convalescent plasma composition and immunological effects in severe patients. J Autoimmun.

[3] Acosta-Ampudia Y, Rojas M, Monsalve DM, Rodríguez Y, Ramírez-Santana C, Anaya J-M (2021). Comment on: nature and dimensions of the systemic hyper-inflammation and its attenuation by convalescent plasma in severe COVID-19. J Infect Dis.

[4] Bandopadhyay P, D’Rozario R, Lahiri A, Sarif J, Ray Y, Paul SR (2021). Nature and dimensions of systemic hyperinflammation and its attenuation by convalescent plasma in severe COVID-19. J Infect Dis.

[5] Esmaeili B, Esmaeili S, Pourpak Z (2021). Immunological effects of convalescent plasma therapy for coronavirus: a scoping review. BMC Infect Dis.

[6] Axfors C, Janiaud P, Schmitt AM, Hooft J, Smith ER, Haber NA (2021). Association between convalescent plasma treatment and mortality in COVID-19: a collaborative systematic review and meta-analysis of randomized clinical trials. BMC Infect Dis.

[7] Rojas M, Anaya J-M (2020). Why will it never be known if convalescent plasma is effective for COVID-19. J Transl Autoimmun.

[8] Casadevall A, Dragotakes Q, Johnson PW, Senefeld JW, Klassen SA, Wright RS (2021). Convalescent plasma use in the USA was inversely correlated with COVID-19 mortality. Elife.

[9] Bastard P, Gervais A, Le Voyer T, Rosain J, Philippot Q, Manry J (2021). Autoantibodies neutralizing type I IFNs are present in ~4% of uninfected individuals over 70 years old and account for ~20% of COVID-19 deaths. Sci Immunol..

[10] Cárdenas-Turanzas M, Ensor J, Wakefield C, Zhang K, Wallace SK, Price KJ (2012). Cross-validation of a Sequential Organ Failure Assessment score-based model to predict mortality in patients with cancer admitted to the intensive care unit. J Crit Care.

[11] Diagnosis and Treatment Protocol for Novel Coronavirus Pneumonia (Trial Version 7). Chin Med J (Engl). 2020;133:1087–95. http://doi.org/10.1097/CM9.0000000000000819. 10.1097/CM9.0000000000000819 PMC7213636 32358325

[12] Tonn T, Corman VM, Johnsen M, Richter A, Rodionov RN, Drosten C (2020). Stability and neutralising capacity of SARS-CoV-2-specific antibodies in convalescent plasma. Lancet Microbe.

[13] Saavedra Trujillo CH (2020). Consenso colombiano de atención, diagnóstico y manejo de la infección por SARS-COV-2/COVID 19 en establecimientos de atención de la salud Recomendaciones basadas en consenso de expertos e informadas en la evidencia. Infect.

[14] World Health Organisation (2020). A minimal common outcome measure set for COVID-19 clinical research. Lancet Infect Dis.

[15] Theel ES, Harring J, Hilgart H, Granger D (2020). Performance characteristics of four high-throughput immunoassays for detection of IgG antibodies against SARS-CoV-2. J Clin Microbiol.

[16] Weidner L, Gänsdorfer S, Unterweger S, Weseslindtner L, Drexler C, Farcet M (2020). Quantification of SARS-CoV-2 antibodies with eight commercially available immunoassays. J Clin Virol Off Publ Pan Am Soc Clin Virol.

[17] Okba NMA, Müller MA, Li W, Wang C, GeurtsvanKessel CH, Corman VM (2020). Severe acute respiratory syndrome coronavirus 2−specific antibody responses in coronavirus disease patients. Emerg Infect Dis.

[18] Rambaut A, Holmes EC, O’Toole Á, Hill V, McCrone JT, Ruis C (2020). A dynamic nomenclature proposal for SARS-CoV-2 lineages to assist genomic epidemiology. Nat Microbiol.

[19] Castañeda S, Patiño LH, Muñoz M, Ballesteros N, Guerrero-Araya E, Paredes-Sabja D (2021). Evolution and epidemic spread of SARS-CoV-2 in Colombia: a year into the pandemic. Vaccines.

[20] Global COVID-19 Clinical Platform: Rapid core case report form (CRF). WHO. 2020. https://www.who.int/publications/i/item/WHO-2019-nCoV-Clinical_CRF-2020.4.

[21] Verbeke G. Linear mixed models for longitudinal data. In: Linear mixed models in practice. Springer; 1997. p. 63–153.

[22] Rao CR (1971). Estimation of variance and covariance components—MINQUE theory. J Multivar Anal.

[23] Team Rs. RStudio: Integrated Development for R. RStudio, PBC, Boston, MA, 2020.

[24] Goldstein H (1986). Multilevel mixed linear model analysis using iterative generalized least squares. Biometrika.

[25] Fitzmaurice GM, Laird NM, Ware JH (2012). Applied longitudinal analysis.

[26] Diggle P, Liang K-Y, Zeger SL (1994). Longitudinal data analysis. New York Oxford Univ Press.

[27] Fitzmaurice G, Davidian M, Verbeke G, Molenberghs G (2008). Longitudinal data analysis.

[28] Cepeda-Cuervo E (2015). Beta regression models: Joint mean and variance modeling. J Stat Theory Pract.

[29] Joyner MJ, Carter RE, Senefeld JW, Klassen SA, Mills JR, Johnson PW (2021). Convalescent plasma antibody levels and the risk of death from Covid-19. N Engl J Med.

[30] Simonovich VA, Burgos Pratx LD, Scibona P, Beruto M V, Vallone MG, Vázquez C, et al. A Randomized Trial of Convalescent Plasma in Covid-19 Severe Pneumonia. N Engl J Med. 2020;:NEJMoa2031304. 10.1056/NEJMoa2031304 PMC7722692 33232588

[31] Shen C, Wang Z, Zhao F, Yang Y, Li J, Yuan J (2020). Treatment of 5 critically ill patients with COVID-19 with convalescent plasma. JAMA.

[32] Duan K, Liu B, Li C, Zhang H, Yu T, Qu J (2020). Effectiveness of convalescent plasma therapy in severe COVID-19 patients. Proc Natl Acad Sci.

[33] Li L, Zhang W, Hu Y, Tong X, Zheng S, Yang J (2020). Effect of convalescent plasma therapy on time to clinical improvement in patients with severe and life-threatening COVID-19: a randomized clinical trial. JAMA.

[34] Avendaño-Solá C, Ramos-Martínez A, Muñez-Rubio E, Ruiz-Antorán B, Malode Molina R, Torres F (2021). A multicenter randomized open-label clinical trial for convalescent plasma in patients hospitalized with COVID-19 pneumonia. J Clin Invest.

[35] Agarwal A, Mukherjee A, Kumar G, Chatterjee P, Bhatnagar T, Malhotra P (2020). Convalescent plasma in the management of moderate covid-19 in adults in India: open label phase II multicentre randomised controlled trial (PLACID Trial). BMJ.

[36] O’Toole Á, Hill V, Pybus OG, Watts A, Bogoch II, Khan K (2021). Tracking the international spread of SARS-CoV-2 lineages B.1.1.7 and B.1.351/501Y-V2 with grinch. Wellcome Open Res..

[37] Naveca FG, Nascimento V, de Souza VC, Corado AL, Nascimento F, Silva G (2021). COVID-19 in Amazonas, Brazil, was driven by the persistence of endemic lineages and P1 emergence. Nat Med.

[38] Foix A, López D, Díez-Fuertes F, McConnell MJ, Martín-Galiano AJ (2022). Predicted impact of the viral mutational landscape on the cytotoxic response against SARS-CoV-2. PLoS Comput Biol.

[39] Li Q, Wu J, Nie J, Zhang L, Hao H, Liu S (2020). The Impact of Mutations in SARS-CoV-2 Spike on Viral Infectivity and Antigenicity. Cell.

[40] Harvey WT, Carabelli AM, Jackson B, Gupta RK, Thomson EC, Harrison EM (2021). SARS-CoV-2 variants, spike mutations and immune escape. Nat Rev Microbiol.

[41] Ray Y, Paul SR, Bandopadhyay P, D’Rozario R, Sarif J, Raychaudhuri D (2022). A phase 2 single center open label randomised control trial for convalescent plasma therapy in patients with severe COVID-19. Nat Commun.

[42] AlQahtani M, Abdulrahman A, Almadani A, Alali SY, Al Zamrooni AM, Hejab AH (2021). Randomized controlled trial of convalescent plasma therapy against standard therapy in patients with severe COVID-19 disease. Sci Rep.

[43] Bajpai M, Kumar S, Maheshwari A, Chhabra K, kale P, Gupta A, et al. Efficacy of Convalescent Plasma Therapy compared to Fresh Frozen Plasma in Severely ill COVID-19 Patients: A Pilot Randomized Controlled Trial. medRxiv. 2020;2020.10.25.20219337.

[44] Gharbharan A, Jordans CCE, GeurtsvanKessel C, den Hollander JG, Karim F, Mollema FPN (2021). Effects of potent neutralizing antibodies from convalescent plasma in patients hospitalized for severe SARS-CoV-2 infection. Nat Commun.

[45] Balcells ME, Rojas L, Le Corre N, Martínez-Valdebenito C, Ceballos ME, Ferrés M (2021). Early versus deferred anti-SARS-CoV-2 convalescent plasma in patients admitted for COVID-19: A randomized phase II clinical trial. PLoS Med.

[46] Libster R, Pérez Marc G, Wappner D, Coviello S, Bianchi A, Braem V (2021). Early high-titer plasma therapy to prevent severe covid-19 in older adults. N Engl J Med.

[47] RECOVERY Collaborative Group (2021). Convalescent plasma in patients admitted to hospital with COVID-19 (RECOVERY): a randomised controlled, open-label, platform trial. Lancet (London, England).

[48] Sullivan DJ, Gebo KA, Shoham S, Bloch EM, Lau B, Shenoy AG (2022). Early outpatient treatment for covid-19 with convalescent plasma. N Engl J Med.

[49] Bajpai M, Maheshwari A, Dogra V, Kumar S, Gupta E, Kale P (2022). Efficacy of convalescent plasma therapy in the patient with COVID-19: a randomised control trial (COPLA-II trial). BMJ Open.

[50] Park H, Tarpey T, Liu M, Goldfeld K, Wu Y, Wu D (2022). Development and validation of a treatment benefit index to identify hospitalized patients with COVID-19 who may benefit from convalescent plasma. JAMA Netw open.

[51] Chang SE, Feng A, Meng W, Apostolidis SA, Mack E, Artandi M (2021). New-onset IgG autoantibodies in hospitalized patients with COVID-19. Nat Commun.

[52] Anaya J-M, Monsalve DM, Rojas M, Rodríguez Y, Montoya-García N, Mancera-Navarro LM (2021). Latent rheumatic, thyroid and phospholipid autoimmunity in hospitalized patients with COVID-19. J Transl Autoimmun.

[53] Savvateeva E, Filippova M, Valuev-Elliston V, Nuralieva N, Yukina M, Troshina E (2021). Microarray-based detection of antibodies against SARS-CoV-2 proteins, common respiratory viruses and type i interferons. Viruses.

[54] Raadsen MP, Gharbharan A, Jordans CCE, Mykytyn AZ, Lamers MM, van den Doel PB (2022). Interferon-α2 auto-antibodies in convalescent plasma therapy for COVID-19. J Clin Immunol.

[55] Fung M, Nambiar A, Pandey S, Aldrich JM, Teraoka J, Freise C (2021). Treatment of immunocompromised COVID-19 patients with convalescent plasma. Transpl Infect Dis.

[56] Rodionov RN, Biener A, Spieth P, Achleitner M, Hölig K, Aringer M (2021). Potential benefit of convalescent plasma transfusions in immunocompromised patients with COVID-19. The Lancet Microbe.

